# Post-traumatic growth in caregivers of children hospitalized in the PICU due to traffic accident: a qualitative study

**DOI:** 10.1186/s12912-023-01213-z

**Published:** 2023-02-23

**Authors:** Zhi Hong Ni, Hai Tao Lv, Jin Hua Wu, Fang Wang

**Affiliations:** Children’s Hospital of Soochow University, No. 92, Zhong nan St, Suzhou, 215025 China

**Keywords:** Caregiver, post-traumatic growth, Accident injury, Qualitative study

## Abstract

**Background:**

Globally, tens of millions of children are hospitalized every year for non-fatal traffic accident injuries, being confronted with an injured child can be extremely stressful for parents. Understandably, a significant level of psychological distress may ensue. Traumatic losses may lead parents to find new insights in life and develop a greater sense of spirituality and strength.

**Method:**

Semi-structured interviews were conducted with caregivers of children who were hospitalized in the pediatric intensive care unit (PICU) with traffic accident injuries at children’s hospitals in China between January and June 2022. Caregivers were selected using a purposive sampling method until no new data were generated (*n* = 24).

**Results:**

We identified eleven sub-themes and four higher-order themes based on these sub-themes: (1) changes in their life philosophy, (2) personal strength enhancement, (3) relationship improvements, and (4) effective responses. The findings of our research contribute to a better understanding of the psychological status of the caregivers of children injured by traffic accidents.

**Conclusion:**

Professionals should guide caregivers from a positive perspective, stimulate their strengths and potential, increase personnel support and communication, promote positive coping, formulate targeted management countermeasures to improve the PTG level of caregivers, and develop strategies to maintain stable mental health and well-being.

## Introduction

Globally, tens of millions of children are hospitalized every year for non-fatal traffic accident injuries [1]. These car accidents often lead to serious bodily injuries [2], such as rib fracture, skull fracture, lumbar fracture, limb fracture, pneumothorax, etc. Serious injuries due to car accidents can lead to lifelong physical disabilities.

Children who are injured in car accidents are usually admitted to the intensive care unit (ICU) for rescue treatment. Medical treatment and professional care are generally required for a period of time.

Following critical injuries, children rely heavily on their parents for physical and emotional support. In the initial acute period, being confronted with an injured child can be extremely stressful for parents. Understandably, a significant level of psychological distress may ensue [3–6]. Between 15%–27% of parents experience moderate-severe depression and anxiety, and 49%–54% report symptoms of acute stress disorder four weeks following their child’s illness or injury [7]. In the post-injury period, parents of children with more severe injuries have higher caregiver burden and distress than those of children with less severe injuries [8].

Children who are injured during traffic accidents tend to have severe injuries. Substantial rehabilitation and care may be required after discharge. These challenges may lead to considerable pressure among the parents and other caregivers.

Although rearing a child who sustained injuries from a traffic accident can result in marked psychological distress for parents, there is increasing evidence that the parents of injured children may demonstrate considerable strength and articulate the positive contributions of their child’s injury to their lives [9]. Traumatic losses may lead parents to find new insights in life and develop a greater sense of spirituality and strength. Bayat (2010) provided evidence supporting spiritual and personal growth as an outcome of care [10].

Post-traumatic growth (PTG) refers to positive psychological changes in self-cognition, life philosophy and interpersonal relationships. Improving the relationship with others, identifying new possibilities in life, strengthening personal spiritual growth and enhancing appreciation of life are the positive changes in coping with adverse events [11]. PTG is closely related to psychological results, such as depression, stress and anxiety. PTG has attracted extensive attention of researchers in the field of healthcare [12, 13].The processing of traumatic events can influence an individual’s beliefs about the world, which may result in their coping with the trauma [14]. Moreover, individuals with positive psychological perspectives are more likely to develop adaptive coping strategies [15].

Research in PTG may offer a potential alternative of a positive perspective on traumatic events. Studies in PTG have explored the possibility of turning parental suffering from adverse events into an opportunity for the parents to uncover positive meaning and effect constructive changes, both of which are important to their well-being [16]. Hefferon, Grealy, and Mutrie (2009) explored the existence of post-traumatic growth in life-threatening physical illness and concluded that PTG is an important but understudied area [17].

The socio-cultural environment plays an essential role in the formation of positive changes [18]. Notably, the Chinese culture places a high value on perfection and achievement [19]. Parents of children with injuries may feel greater stress and isolation due to the social and behavioral problems associated with children with disabilities. This study aimed to explore the evidence of PTG in Chinese mothers of children with injuries from traffic accidents.

Limited empirical evidence relating to caregivers of children hospitalized in the PICU with injuries following traffic accidents exists in the literature on PTG. Hence, our study aimed to research this aspect.

Therefore, this study aimed to develop an understanding of healthcare providers pay more attention to this specific subgroup of individuals, and assist them in improving their health and quality of life. This understanding can support the provision of psychological support to caregivers of children injured in traffic accidents.

## Methods

### Design

This study used a qualitative design to analyze PTG in caregivers of children with traffic accident injuries who were hospitalized in the PICU. All interviews were conducted between January and June 2022 in the XX. Purposive sampling was used to enroll the caregivers of children hospitalized in the PICU with injuries following a traffic accident.

### Participants

As mentioned, a purposive sampling method was used to select the participants. All the participants were carers of children injured in a car accident. The main diagnosis included rib fracture, skull fracture, lumbar fracture, limb fracture, pneumothorax, etc. The inclusion criteria for the children were as follows. (1) The child survived the car accident. (2) The child’s condition was stable. (3) The child was aged between 5 and 16 years. (4) One month after the accident injury. The exclusion criteria included (1) children who were unconscious and unable to communicate and (2) children in an unstable and life-threatening condition.

The caregivers’ inclusion criteria were as follows. (1) Adults aged 18 years and over. (2) A child participant’s parent. (3) Normal communication and expression skills. Caregivers with a diagnosed mental illness were excluded from this study.

The general characteristics of the children and their caregivers are listed in Tables 1 and 2, respectively.

**Table 1:** Demographic data of the child suffer traffic accident injury

**Variable**	**N**	**F (%)**
Gender		
Male	13	54.2
Female	11	45.8
Age (year)		
5-8	5	20.8
9-12	12	50.0
13-16	7	29.2
Injury types		
Rib fracture	5	20.8
Skull fracture	7	29.2
Lumbar fracture	5	20.8
Limb fracture	4	16.7
Pneumothorax	3	12.5

**Table 2:** Demographic data of the caregivers

**Variable**	**n**	**F (%)**
Education		
Middle school	5	20.8
Junior college	9	37.5
University	10	41.7
Age		
<30	6	23.1
31-40	7	34.6
1	11	42.3
Occupation		
Unemployed	4	15.4
Company worker	7	30.8
Agricultural worker	5	19.2
Office clerk	8	34.6
Residence		
City	10	42.3
County	14	57.7
Caregiver		
Mother	18	61.5
Father	6	38.5

### Data collection

Interviews were conducted in a quiet consultation room at the hospital. The qualitative data collection method included semi-structured, face-to-face interviews. A senior researcher (NZH) performed the interviews and also trained the less experienced co-workers. NZH is an experienced PhD-qualified nurse. Moreover, all the researchers in this study are experienced in performing qualitative research. To develop the semi-structured interview, we consulted five ICU nurses and referred them to relevant systematic reviews in the literature [20, 21]. Initially, a preliminary interview was conducted with the five caregivers. The data from the preliminary interviews were not included in this study but were used to modify the interview structure according to the preliminary outcomes. The final interview used in this study included the items as follows.

The caregivers were asked:

- (1) What are your experience and feelings of your child being admitted to the ICU for treatment after being injured? (2) What psychological changes did you experience during this time? (3) In the process of experiencing a family illness, have you made any positive changes? (4) What are some of the difficulties and pressures you have experienced during this time, and how do you deal with them? (5) What are your plans for the future? (6) If you encounter caregivers with similar experiences to you, what advice do you have for them?

To capture the parents’ lived experiences of caring for their children with injuries following a traffic accident in real-time, we conducted one-on-one interviews with the caregivers 1 month after the injury of their children. Only the caregiver and interviewer were present during the interviews. No one else was allowed in the interview room. Each interview lasted 35–55 min. The interviewer first introduced herself to the caregivers and gained their trust. During the interview, some drinks and food were provided. If the caregivers were tired, they were given time to rest. During the interview, the nurses took care of the children. We continuously collected data until no new events occurred, thereby achieving data saturation [22] ^.^Audio recordings were used to collect data and field notes were created after each interview.

### Data analysis

For the qualitative content analysis [23]. the interviews were first transcribed word for word, and then the interview notes were compiled. Data analysis was conducted using the NVIVO software (QST International, Cambridge, MA, USA). The investigators read the transcripts to familiarize themselves with the data and then extracted the most relevant words and phrases to describe the caregiver’s experiences in caring for their injured child. The investigators read all transcripts and extracted sentences that conveyed the most meaningful information regarding the caregiver’s experiences and needs. This was followed by the preparation of coding sheets, grouping of the data, and creation and abstraction of the categories. Codes were used for the various descriptions. Data categorization was performed multiple times by the investigators, who worked closely together until the four main categories were identified. As a confirmatory test, the four categories were shown to caregivers who all agreed that the results accurately represented their experiences [23].

### Ethical considerations

This study was conducted in accordance with the Declaration of Helsinki. We confirm that all methods were performed in accordance with the relevant guidelines and regulations. Ethical approval was approved by the ethics committee of Children’s Hospital, Soochow University, Suzhou City, Jiangsu Province, China (approval no., 2021ks001). Informed consent was signed by each participant before being interviewed and was coded to maintain anonymity. Data were stored in a locked cabinet and all electronic copies were password protected and could only be accessed by the research team.

## Results

Through data analysis, we identified the following four themes: (1) changes in life philosophy, (2) personal strength enhancement, (3) relationship improvements, and (4) effective responses (Table 3). Each theme is described below with supporting quotes from the participants.

**Table 3:** Superordinate and sub-themes identified in the analysis

Themes	Sub-themes
(1) changes in philosophy of life	*Appreciation of life*
	*Establish priorities in life*
	*Uncertain future*
(2) personal strength enhancement	*Tap advantages and potentials*
	*Enhanced sense of responsibility*
	*Enhanced sense of self-reliance*
(3) improving relationships with others	*“Self serving and altruistic” behavior*
	*Benefit from interpersonal communication*
(4) effective response	*Set hope*
	*self-consolation*
	*Learn to adjust themselves*

### Changes in life philosophy

#### Appreciation of life

After a child sustained injuries following a traffic accident, most of the caregivers indicated they would have different plans for their lives.

‘Nothing is better than living safely. I hope everyone in the family is safe. No one should have an accident.’ Caregiver #5

‘Life is fragile. We should cherish life and health.’ Caregiver #11

Some caregivers felt that they should cherish their families more and show reverence for life.

‘At the door of ICU, I see joys and sorrows every day. When fate comes, it will not give you time to prepare, nor will it give you the slightest chance!’ Caregiver #2

#### Establish priorities in life

The caregivers said that after the child sustained injuries from a traffic accident, they realized what the really important things in life are and changed their priorities. Some caregivers feel that the most important thing is to have family members to accompany and support them.

‘The child recovered well after the operation. I stopped all my work. I traveled with him everywhere. I invited his friends to visit. It’s really nice to have everyone with him.’ Caregiver #8

‘This time I must reconsider my life. I won’t work anymore. I will first consider my daughter’s health. I told my boss that I’ll put down my work for the time being.’ Caregiver #6

Some caregivers also explained that they had changed their work plans to prioritize the health of their child, which is now the most important.

‘I suddenly felt that my child had studied too hard in the past. I wanted to give him a good rest in the future to stop him from being so tired.’ Caregiver #12.

#### Uncertain future

Due to the unpredictability and difficulty in treating children after car accidents, the caregivers also have shared a sense of uncertainty about their child’s treatment, rehabilitation, and prognosis.

‘The child’s injury will affect their limb function. Not easy to recover… ‘ Caregiver #1.

‘The child is undergoing rehabilitation treatment, I don’t know what improvement my child will achieve in the future through rehabilitation.’ Caregiver #16

The high medical expenses associated with treating children with injuries following a traffic accident can lead to heavy economic burdens for their families. The caregivers feel helpless about their future. For rural families or self-funded families, their sense of powerlessness in the face of the future is even more obvious.

‘This time, all the money for my son’s injury treatment was borrowed from relatives and friends. Only 50% of my child’s medical expenses can be reimbursed from insurance. I think about how to pay for my child’s treatment in the future every day (sigh).’ Caregiver #9

### Personal strength enhancement

#### Enhanced strengths and potential

Some caregivers uncover excellent qualities in themselves, such as optimism, firmness, calmness, and persistence. After their child was injured in a car accident, even when the child was critically ill, these caregivers believe that everything should be good.

‘My life has encountered such a big disaster, I must be rational and calm when considering problems and control my emotions. I am the father of the child, so I must support the whole family!’ Caregiver #10

“The doctors asked me to sign consent when they rescued my children. I was very afraid at that time. I know that my hesitation is useless. I must make a prompt decision and not delay the rescue of my child.’ Caregiver #4.

#### Enhanced sense of responsibility

The caregivers have a strong sense of responsibility in taking care of their children. They refer to the responsibility of actively guarding the family.

‘I have been taking care of the child since he was injured in a car accident. He is my child, and I can’t leave him when he is ill. No one at home can help me, and I won’t complain about them.’ Caregiver #14

‘My child’s mother was struck and weakened, so I must take good care of my child, deal with everything at home well, and make others feel at ease. I think I can’t stand back anymore. I should take responsibility!’ Caregiver #7

#### Enhanced sense of self-reliance

Over the prolonged course of treatment, the caregivers’ sense of self-reliance is significantly enhanced.

‘The child’s injury cannot be recovered in a short time. It’s not enough to rely on others all the time. My relatives are busy with their work, and they have their own businesses. I have to rely on myself!’ Caregiver #16

The caregiver’s sense of self-reliance may also increase due to insufficient family support.

‘My mother-in-law is in poor health. My husband has to go to work and has a lot of work pressure. No matter how much pressure I have, I can’t talk to them. I can’t find anyone else to help. I can only bear all the hardships alone.’ Caregiver #15

### Relationship improvement

#### Reciprocal altruistic behaviors

The caregivers showed mutually beneficial altruistic behaviors based on their own needs. After the experience of having to cope with an injured child, the caregivers became more compassionate and were more willing to help others. They also reflected on the improvement of their consciousness to choose to do the right thing.

‘Sometimes I think that if I do something good, I’m helping my daughter... I think that if I help others, there will be good fortune for my daughter.’ Caregiver #17

Some caregivers established a WeChat social group for the family members of ICU patients. Through this platform, the caregivers provide mutual assistance and support to each other and the group also advocates support for family members and peers.

‘Now I’m willing to help if I can help others. I think helping others may reduce my son’s suffering. That’s what I think. Helping others at critical moments can bring warmth to others.’ Caregiver #21

#### Benefits from interpersonal communication

The caregivers appreciated the help from professionals and the mutual support between patients’ families. They benefited from these interpersonal interactions. Notably, the caregivers expressed and affirmed their gratitude to the professionals who provided treatment to their children.

‘My child was transferred by ambulance to this hospital. If the medical staff did not treat my child on time, my child may have no hope (crying). I am very grateful to these doctors and nurses who helped me so.’ Caregiver #18

‘My child went out for a CT examination and other family members helped me take care of my child. They were all very good.’ Caregiver #20

‘Caregivers sometimes talk about their experiences during treatment. I ask them about some successful rehabilitation treatment methods and experiences. I benefited a lot from talking to them.’ Caregiver #22

### Effective response

#### Hope

The family members of ICU patients constantly adjusted their emotions and sought hope during the difficult treatment period.

‘I saw that there was a patient who was cured and transferred out, and I thought we had hope. She was in the same situation as my daughter. She was transferred out of the room to the general ward. I think my daughter will be as good as her.’ Caregiver #19

‘In recent days, several children in ICU in succession have been out of danger. I’m really happy for them. Originally, their situations were very dangerous!’ Caregiver #3

#### Self-consolation

The caregivers changed the standards for measuring things and reshaped a positive attitude through self-suggestion.

‘Today, the doctor told me that my child’s walking ability may be affected in the future. I think it’s better than limb paralysis. We still have hope. We can work together to do rehabilitation training. I’ll psychologically prepare myself and self-consolidate.’ Caregiver #24

#### Learn to adjust

Most of the caregivers in this study indicated that they have learned to maintain psychological balance through self-adjustment when dealing with difficulties or when they encounter negative emotions.

‘My friends say that I take try too hard to care for my children. In fact, I have a good attitude. I relieve myself. When I’m sad, I cry, or find someone to confide in and share from my heart. I’ll just vent.’ Caregiver #24

‘I feel very anxious myself. The child’s recovery after the injury may be a slow recovery process. I told myself to be patient and wait patiently for her to slowly get better.’ Caregiver #23

## Discussion

This study explored the PTG among caregivers of children injured following a traffic accident who were hospitalized in the PICU. Our results showed that the caregivers developed PTG after the injury of their child and during hospitalization in the PICU. This finding is consistent with the results of other studies [24, 25]. Positive psychological change is the main embodiment of the PTG among the caregivers of children with traffic accident injuries.

In this study, the caregivers demonstrate some positive psychological changes after experiencing adverse events, including changes in life philosophy, personal strength enhancement, and relationship improvement. There were also positive psychological changes among the family members of patients in this study, which concurred with the PTG theory constructed by Tedeschi et al. (2017) [26] .

This positive self-change phenomenon has been confirmed in other relevant studies [25, 27]. PTG is a process, which is affected by many factors, including event-related factors, and personal and environmental systems [28]. PTG can also be regarded as a result. The result of growth does not mean that psychological pressure and existing difficulties disappear. Instead, family members become stronger and have a deeper understanding of life by fighting against adversities. The caregivers have a strong sense of responsibility in taking care of their children. After his child was injured and hospitalized, the father felt that he should take care of the child and become more responsible. It is suggested that professionals can help caregivers better adjust psychologically and improve their PTG level by increasing their interpersonal resources, uncovering their strengths and potentials, and guiding them to pay attention to the meaningful things in life.

Accepting uncertainty is an important factor in promoting the growth of caregivers of children injured after a car accident. In contrast to the PTG theory, the theme of “uncertain future” in this study reflected that the growth experience of new possibilities among the family members is not prominent [29]. The object of this study was the family members of the children injured following a traffic accident. During the children’s stay in the PICU, family members often devote a lot of time dealing with their children’s injuries. Hence, their social activities reduced, and some caregivers even experienced social behavior withdrawal. No extra energy to cultivate new interests or replan life was also relatively common. Therefore, professionals should take the initiative to care for the family members and help as necessary to increase their sense of control over the situation. In addition, by organizing group activities and applying the focus shift method [30], caregivers can avoid overthinking about the adverse situation. The professionals must actively guide the caregivers to adapt to the situation, encourage them to rebuild their planned life, and increase their tolerance for uncertainties, all of which are conducive to PTG.

Effective coping is an important sign that the family members of children injured in a car accident have grown. In this study, the caregivers of children with car accident injuries demonstrated various effective coping styles. For example, setting hope, self-consolation, learning to adjust themselves, etc. These are conducive to the caregivers enhancing their confidence in dealing with the prognosis of the disease, helping to maintain a psychological balance, and shaping a positive attitude [31]. This study showed that adopting positive and effective coping styles ensured that the individual’s growth experience during traumatic events is enhanced. This finding is consistent with the results of other studies [32, 33]. In this study, the caregivers mentioned that when the nearby patients were getting better after treatment, they felt hopeful. This hope increased their confidence in their child’s treatment. The research on the PTG of parents of child patients showed that the parents’ perception of hope in the care process can promote their PTG. Hence, having hope is important for ensuring a positive attitude change [34].

The family members of the patients in this study also maintained a good state of mind by venting their emotions, comforting themselves, and encouraging themselves to actively face difficulties. Research on PTG confirms that a positive coping style pointing to the future is an extremely valuable psychological resource for patients when dealing with trauma, which is conducive to their reconstruction of social functions and reintegration into society [35]. It has been suggested that professionals should fully evaluate the coping styles of caregivers and family members of their patients, to help them build hope, guide them to adopt positive coping strategies, increase their self-efficacy, and further promote their PTG through relevant awareness and education, including the citing of successful cases, and shared-family activities and experiences.

### Limitations

This study aimed to explore the post-traumatic growth among the caregivers of children hospitalized in the PICU due to traffic accidents. This study has several limitations. First, the findings of this study reflect only the experiences of 24 caregivers with children hospitalized in the PICU due to traffic accidents in China, who voluntarily participated. Second, this research focused solely on the perceptions, thoughts, and feelings of these 24 caregivers and did not take into account the experience of the siblings, or the patients. Third, the interview sample consisted of caregivers who were aware that they would need to articulate their post-traumatic growth experiences associated with the care of their children who were hospitalized in the PICU due to traffic accidents, Logistical restraints, including time, may have precluded a more in-depth analysis and integrated presentation of the large amount of data collected for this study.

### Clinical implications

It may be helpful to encourage caregivers to adopt an effective way to deal with their problems and maximize a strong support network from family, friends, and helping professionals to provide emotional or practical support.

## Conclusion

This study explored the caregiver experience of PTG following a traffic accident that resulted in an injured child being hospitalized in the PICU in Jiangsu Province, China. Changes in life, personal strength enhancement, relationship improvement, and effective response development portray the caregiver experience of PTG following a traffic accident in which their child is injured and hospitalized in the PICU.

Professionals should guide caregivers from a positive perspective, stimulate their strengths and potential, increase personnel support and communication, promote positive coping, formulate targeted management countermeasures to improve the PTG level of caregivers, and develop strategies to maintain stable mental health and well-being.

## Data Availability

The data that support the findings of this study are available from the corresponding author upon reasonable request.
